# Pathological Gambling: The Impact of Early Initiation and Sociodemographic Predictors

**DOI:** 10.1192/j.eurpsy.2026.10837

**Published:** 2026-07-31

**Authors:** R. Peric, O. Sbutega FIlipović, B. Velickovic, M. Gojkovic, M. Purić, M. Gostiljac

**Affiliations:** 1 Special Hospital for Addiction Disorder; 2 Clinic of Psychiatry, University Clinical Centre of Serbia, Belgrade, Serbia

## Abstract

**Introduction:**

Pathological gambling represents one of the more prevalent health disorders associated with addictive behaviors. It exerts a considerable impact on an individual’s social functioning, community integration, and economic stability. Studies indicate that early exposure to gambling is correlated with increased frequency of gambling behavior, a more severe clinical presentation, and more pronounced negative consequences. In addition, the widespread availability and accessibility of betting shops play a significant role in both the earlier onset of gambling and the intensification of its adverse effects. Serbia currently ranked second in the number of betting shops per capita.

**Objectives:**

The objective of this study is to highlight the importance of sociodemographic factors with earlier onset of gambling and their impact on the course and consequences of the disease itself.

**Methods:**

This retrospective study was conducted in December 2023. Data was collected from the Special Hospital for Addiction Disorder in Belgrade, Serbia and included 200 patients who were on treatment in the hospital from January 2022 to June 2023. Statistical analyses included the chi-square test, Fisher’s exact test, and logistic regression.

**Results:**

Of the total of 200 patients, 97.5% were male, with the most common age range being 20 to 40 years. The results of this study showed that male gender (χ^2^ = 16.6, p = 0.002) and younger age (χ^2^ = 105.6, p < 0.001) were significantly associated with earlier onset of gambling. The establishment of emotional connections (χ^2^ = 28.6, p = 0.005) and the use of psychoactive substances (χ^2^ = 20.7, p = 0.008) also show a statistically significant association with an earlier onset of gambling. Early onset of gambling has also been shown to be a significant predictor of the emergence of suicidal ideation as well as suicidal attempts themselves.(χ^2^ = 15.9, p = 0.043). Substance use was further correlated with alcohol consumption (χ^2^ = 7.1, p = 0.029), suicidal thoughts (χ^2^ = 100.1, p < 0.001), and suicide attempts (χ^2^ = 99.9, p < 0.001). Older participants were more likely to report gambling expenditures disproportionate to their income (χ^2^ = 23.5, p = 0.023)

**Conclusions:**

The study showed that more frequent and earlier onset of gambling is associated with male youth as well as younger generations. The use of psychoactive substances is one of the key factors that makes the earlier onset of gambling more difficult than the development of the disease. Future research is necessary to clarify the specific predictors and risk factors that influence both the onset and the progression of gambling-related disorders.

**Disclosure of Interest:**

None Declared

